# Unilateral renal cystic disease

**DOI:** 10.4103/0971-4065.65310

**Published:** 2010-04

**Authors:** N. A. Choh, M. Rashid

**Affiliations:** Department of Radiodiagnosis, Govt Medical College, Srinagar, India

Unilateral renal cystic disease of kidney is a non-familial and non-progressive disorder, characterized by replacement of the renal parenchyma by a cluster of multiple cysts with a normal contralateral kidney. This rare condition is not related to autosomal dominant polycystic kidney disease; as such, hepatic and pancreatic cysts are not seen. We report a case of unilateral renal cystic disease in an elderly male documented by CT and serial ultrasound examinations.

**Figure 1:**
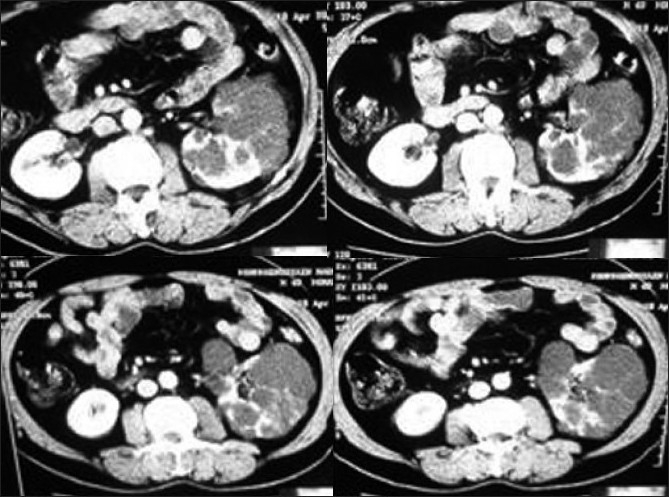
CECT abdomen shows multiple simple cysts replacing the left renal parenchyma with normally enhancing intervening parenchyma. Radiological picture was stable on follow-up

An elderly asymptomatic male (with normal renal function tests and urine examination) was found to have multiple cysts in left kidney during a routine ultrasound examination with thinned out intervening parenchyma; the right renal parenchyma was normal in size and echotexture. There was no evidence of hepatic or pancreatic cysts. Screening of family members did not reveal any evidence of autosomal dominant polycystic kidney disease. Contrast enhanced CT revealed multiple clustered cysts in left kidney, with normal enhancement of the intervening parenchyma [Figure 1]. The right kidney was normal without any cyst. Unilateral renal cystic disease was presumed, and patient put on follow-up. Serial ultrasounds over two years have documented the stability of the left renal cystic lesions, confirming the diagnosis.

Unilateral renal cystic disease, also called unilateral polycystic kidney disease, localized cystic disease of kidney, and segmental polycystic kidney, is characterized by replacement of renal parenchyma, either total or localized to a portion of kidney, by multiple cysts.[1–4] This is distinct from ADPKD, which is bilateral with involvement of both cortex and medulla. In children, ADPKD can have asymmetric onset; in these cases long term follow-up and screening of family members can make the distinction from unilateral cystic disease. The extrarenal manifestations of ADPKD (hepatic and pancreatic cysts, cerebral aneurysms) are not seen in unilateral renal cystic disease. Nephrolithiasis and hyperattenuating cysts are also generally seen in ADPKD.[4,5]

When unilateral cystic disease of kidney is focal, it may be confused with a cystic neoplasm like multilocular cystic nephroma or cystic RCC. Careful analysis of multiple sequential images reveal a continuum of cysts in localized cystic disease of kidney with normal intervening parenchyma as compared to focal encapsulated mass in case of cystic neoplasms with compression of adjacent renal parenchyma.[1,4]

The renal function is preserved with near normal concentration of contrast by the affected kidney. This allows distinction from multicystic dysplastic kidney (an entity seen in infants and children) in which the intercystic parenchyma is dysplastic and non functional; the dysplastic core in MCDK may show some enhancement, but has a different appearance from the normal renal tissue.[1–5]
